# High-Efficient Generation of Induced Pluripotent Stem Cells from Human Astrocytes

**DOI:** 10.1371/journal.pone.0015526

**Published:** 2010-12-09

**Authors:** Sergio Ruiz, Kristen Brennand, Athanasia D. Panopoulos, Aída Herrerías, Fred H. Gage, Juan Carlos Izpisua-Belmonte

**Affiliations:** 1 Gene Expression Laboratory, The Salk Institute for Biological Studies, La Jolla, California, United States of America; 2 Laboratory of Genetics, The Salk Institute for Biological Studies, La Jolla, California, United States of America; 3 Center of Regenerative Medicine in Barcelona, Barcelona, Spain; Wellcome Trust Centre for Stem Cell Research, United Kingdom

## Abstract

The reprogramming of human somatic cells to induced pluripotent stem (hiPS) cells enables the possibility of generating patient-specific autologous cells for regenerative medicine. A number of human somatic cell types have been reported to generate hiPS cells, including fibroblasts, keratinocytes and peripheral blood cells, with variable reprogramming efficiencies and kinetics. Here, we show that human astrocytes can also be reprogrammed into hiPS (ASThiPS) cells, with similar efficiencies to keratinocytes, which are currently reported to have one of the highest somatic reprogramming efficiencies. ASThiPS lines were indistinguishable from human embryonic stem (ES) cells based on the expression of pluripotent markers and the ability to differentiate into the three embryonic germ layers *in vitro* by embryoid body generation and *in vivo* by teratoma formation after injection into immunodeficient mice. Our data demonstrates that a human differentiated neural cell type can be reprogrammed to pluripotency and is consistent with the universality of the somatic reprogramming procedure.

## Introduction

The developmentally committed identity of somatic cells can be reverted to a pluripotent state through different reprogramming approaches. Among these methodologies, pluripotency is achieved by somatic cell nuclear transfer into enucleated unfertilized oocytes, cell fusion of differentiated cells with embryonic stem (ES) cells or treatment of differentiated cells with extracts derived from pluripotent cells [reviewed in 1]. More recently, it has been reported that the induction of pluripotency in somatic cells can be achieved by the expression of defined transcription factors [2]–[6], including either the combination of Oct4, Sox2, Klf4 and cMyc [2]–[5], although the latter was found to be dispensable [7], or Oct4, Sox2, Nanog and Lin28 [6]. Induced pluripotent stem (iPS) cells are epigenetically and functionally similar to ES cells [2]–[6], although studies comparing ES and iPS cells continue to more precisely examine the equivalence of these cell types. While the molecular mechanisms underlying the process of reprogramming remain obscure, recent reports indicate that classical hallmarks of malignancy such as inactivation of the p53 pathway [8]–[10] or silencing of the *ink4/arf* locus [11] leading to immortalization [12] are shared between somatic cell reprogramming and cell transformation.

In the last few years, rapid progress has been made towards improving the efficiency of iPS cell generation, development of integration-free strategies or substitution of some reprogramming factors with other proteins or chemical compounds [13]–[17]. Though initial reports relied on the use of retroviral or lentiviral delivery systems to introduce the reprogramming transcription factors [2]–[6], induction of pluripotency can now be achieved with plasmid transfection [13], non-integrative episomal vectors [14], Piggy-Bac transposition [15], self-excisable vectors [16] or by the delivery of reprogramming proteins [17]. The universality of the process has been demonstrated by the generation of iPS cells from different species [18]–[20], as well as from different sources of somatic cells including fibroblasts [2], CD34+ cells [21], adipose cells [22], HUVEC cells [23], keratinocytes [24], neural stem cells [25] or hepatocytes [26].

Here, we report the generation of iPS cells from human astrocytes (ASThiPS) with a similar efficiency to keratinocytes, one of the human somatic cell types with the highest reported reprogramming efficiency to date. ASThiPS cells show a pluripotent gene expression signature, display an ES-like cell cycle profile, differentiate into the three germ layers *in vitro* and *in vivo,* and generate multiple neuronal cell lineages following directed differentiation.

## Results

### Astrocytes can be reprogrammed to pluripotency with high efficiency

**Figure 1:**
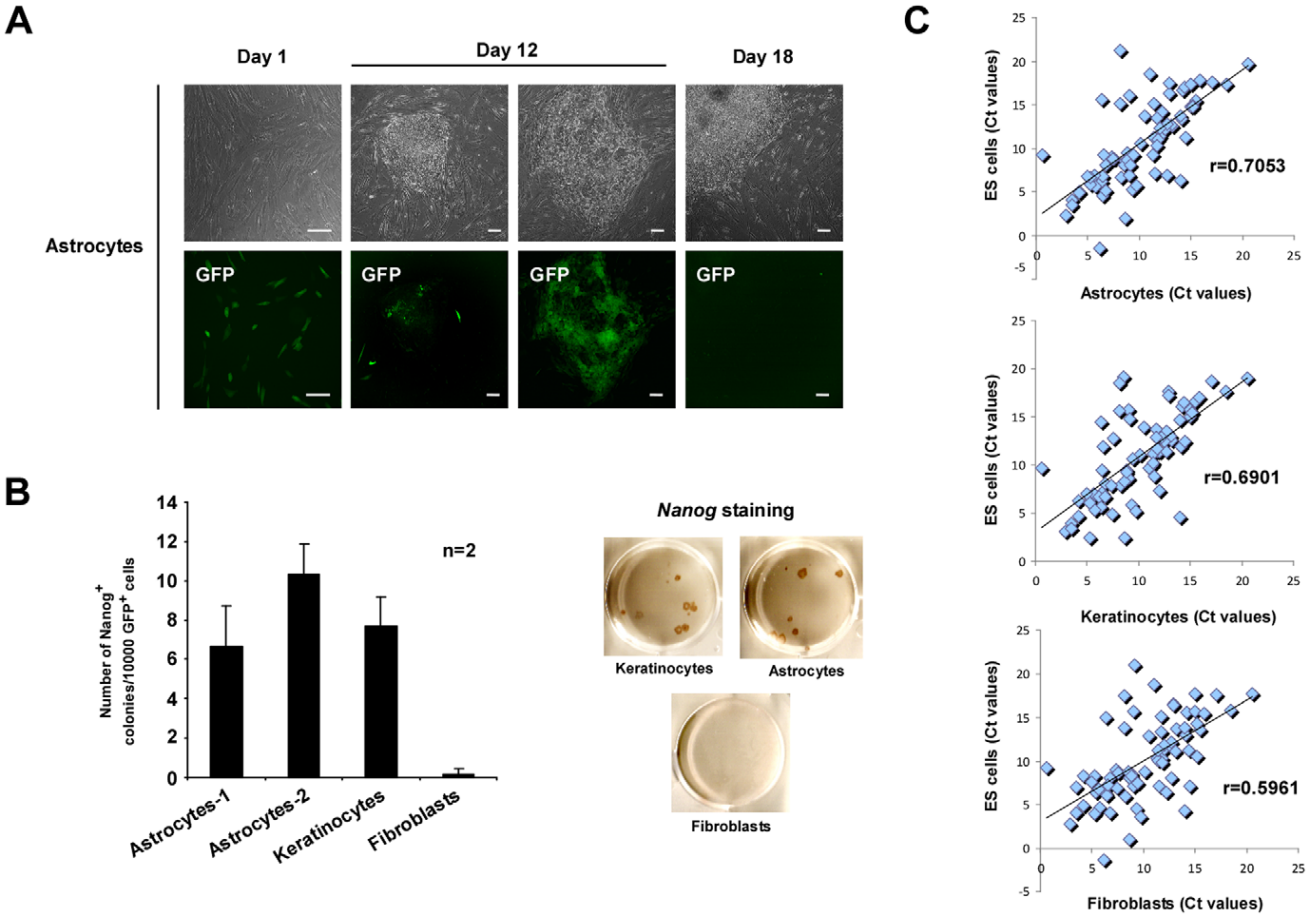
Highly efficient somatic cell reprogramming from astrocytes. (A) Morphological changes and GFP expression in OSKC-infected astrocytes during the reprogramming process at 1 (first column), 12 (second and third columns) and 18 (fourth column) days after the initial infection. Note the efficient GFP silencing in cells with hES cell-like morphology (second column) versus transformed non-hES cell morphology cells (third column). Scale bar: 50 µM. (B) Absolute reprogramming efficiencies, observed for astrocytes (two different sources of astrocytes were used as indicated), fibroblasts and keratinocytes, normalized to the number of GFP positive cells seeded, (left graph). A representative example of Nanog-stained colonies is shown in the right panels. Uninfected cells were used as a negative control for all experiments (data not shown). n = number of independent experiments with 3 biological replicates each. All error bars depict the SEM. (C) Graphical representation of the Ct values normalized to the expression of the GAPDH housekeeping gene between the different groups of values in the noted samples. r = Pearson coefficient.

We investigated the potential of human astrocytes to reprogram into hiPS cells following retroviral transduction with Oct4, Sox2, Klf4 and cMyc (OSKC) transcription factors (Figure 1). We also included a retrovirus encoding GFP to evaluate the efficiency of transduction and visualize exogenous transgene silencing [2]–[6]. Similar reprogramming experiments were carried out in parallel with human keratinocytes, a somatic cell with a high reprogramming efficiency [24] and fibroblasts, a cell type with significantly lower reprogramming efficiency [24]. Two serial spinfections of keratinocyte, fibroblast or astrocyte cultures resulted in over 90%, 50% and 40% of infected cells, respectively (Figure 1A, left column and data not shown). To evaluate the efficiency of reprogramming, identical numbers of GFP^+^ cells were plated onto mouse embryonic fibroblasts (MEFs). Twelve days after the first infection, we started to observe the appearance of morphological hES-like colonies, coinciding with transgene silencing (Figure 1A, second column). However, we also detected partially reprogrammed colonies with non-hES morphological phenotypes, where transgene silencing, based on GFP expression, did not occur (Figure 1A, third column). Finally, eighteen days following the initial infection, we either fixed/stained or manually picked the hiPS cell colonies obtained for further culture and characterization (Figure 1A, right column). Reprogramming efficiency was defined as the number of Nanog positive colonies per 10000 GFP^+^ cells seeded on MEFs. We observed a similar reprogramming efficiency for human astrocytes and keratinocytes, which was much higher when compared to fibroblasts (Figure 1B). Similar reprogramming experiments performed using alkaline phosphatase staining to evaluate pluripotent colony formation showed comparable results (data not shown). To better understand why astrocytes reprogram more efficiently than other cell types such as fibroblasts, we performed a real-time analysis of the expression of genes involved in different aspects of stem cell biology (for a complete description see Material and Methods and Table S1, which contains the total list of genes analyzed) in the H9 hES cell line, astrocytes, keratinocytes and fibroblasts. Using a Pearson correlation as a distance measure between the different sets of values we observed that both, keratinocytes and astrocytes, are closer than fibroblasts to hES cells (Figure 1C), which may contribute to the higher reprogramming efficiency of astrocytes.

### Characterization of ASThiPS cell lines

**Figure 2:**
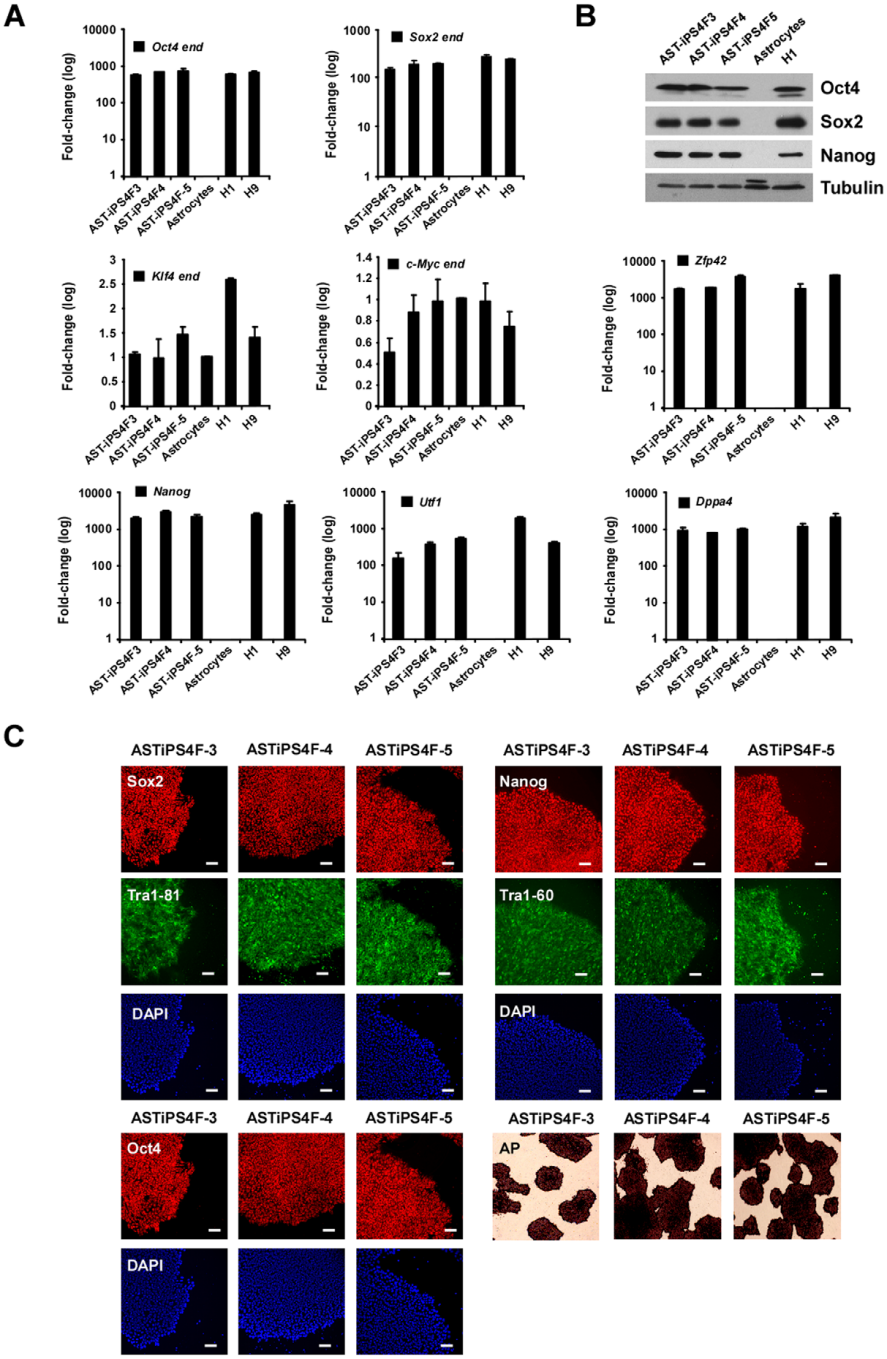
ASThiPS cell lines express pluripotent markers. (A and B) Analysis of the ASThiPS cells lines showing the relative expression of the endogenous levels for the annotated genes by real-time PCR (A) or the total Oct4, Sox2, and Nanog protein levels by western blot (B) compared to hES cells and the somatic cells of origin. The relative gene expression levels of Klf4 and c-Myc are represented in linear scale, whereas those for the rest of genes are represented in log scale. Data are shown as the relative averages ± SEM calculated from two biological replicates analyzed in triplicate. (C) Immunofluorescence analysis of the indicated pluripotent markers in the different ASThiPS cell lines. DAPI was used to visualize nuclei. Alkaline phosphatase (AP)-staining was also performed for each of the ASThiPS cell lines. Scale bar: 50 µM.

We manually picked several colonies of hiPS cells derived from astrocytes (ASThiPS) and established three independent lines for further analysis (Figure 2A). All ASThiPS cell lines showed permanent silencing of the reprogramming transgenes, and displayed expression levels of the endogenous reprogramming genes that were comparable to hES cells (Figure 2A and data not shown; for a complete description of the primers used, see Table S2). Moreover, the expression of several stem cell markers such as Nanog, Utf1, Dppa4, or Zfp42, was indistinguishable from hES cells (Figure 2A, for a complete description of the primers used, see Table S2). We also analyzed several pluripotency markers at the protein level by immunoblotting (Figure 2B; for a complete description of the antibodies used, see Table S3) and immunofluorescence (Figure 2C, Table S3), and validated that all ASThiPS cell lines resembled hES cells in terms of pluripotent gene expression. ASThiPS cell lines were maintained for at least 20 passages without a loss of pluripotency and all stained positive for alkaline phosphatase (AP) activity (Figure 2C and data not shown).

**Figure 3:**
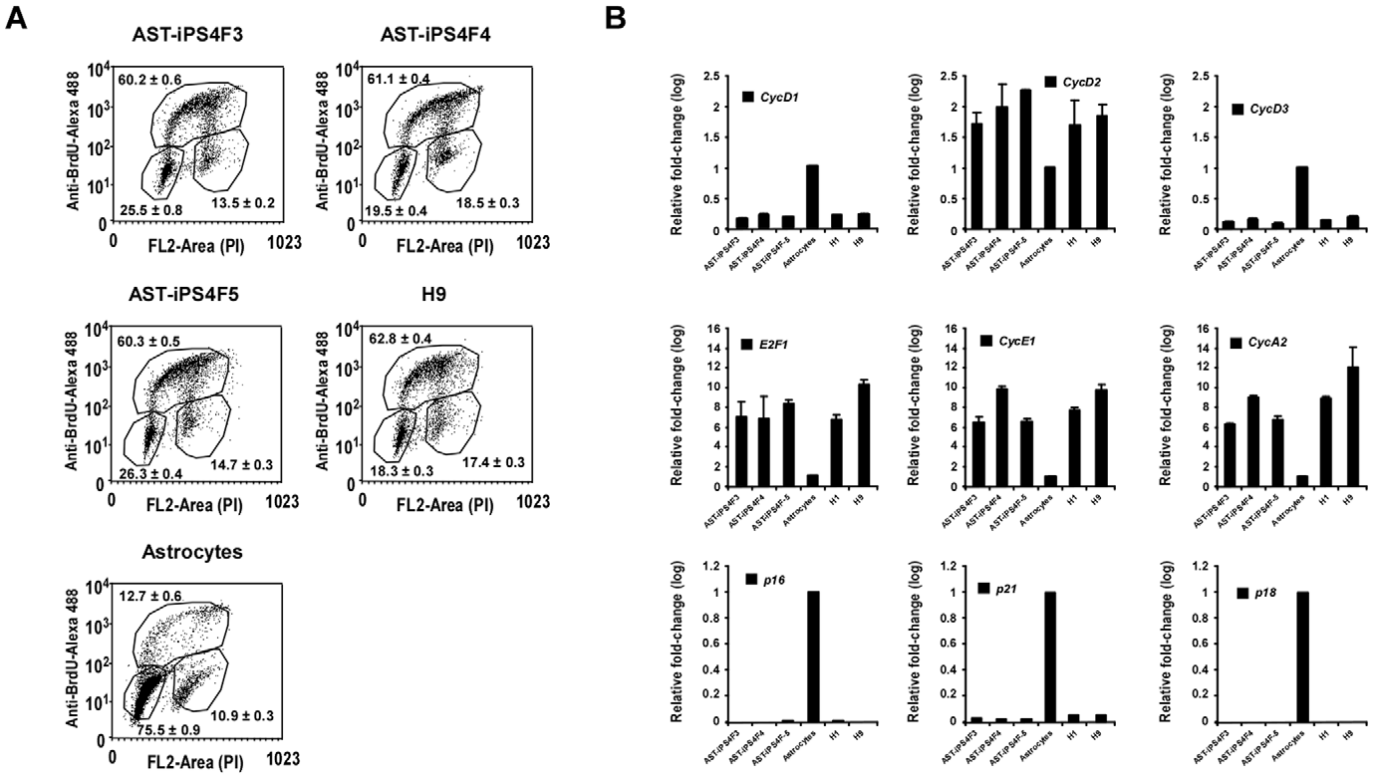
ASThiPS cells acquired the stem cell cycle signature observed in hES cells. (A) BrdU incorporation analyzed by flow cytometry in ASThiPS cell lines, astrocytes and H9 cells. Represented in each dot plot are the percentages of cells in G1 phase (population in the lower left corner), S phase (upper region) and G2 phase (population in the lower right corner). 10,000 cells were represented in each dot plot and the averages ± SEM were calculated from two biological replicates analyzed in triplicate. (B) Analysis of the indicated cell cycle genes, by real-time PCR in ASThiPS cell lines, the parental cells of origin and hES cells. Data in are shown as relative average fold change ± SEM from two biological replicates analyzed in triplicate.

hES cells have a cell cycle signature structure characterized by a very short G1 phase and a high percentage of cells in S phase [27]. We found that the characteristic stem cell cycle signature of hES cells is acquired in all the ASThiPS cell lines and differs greatly from the cell cycle profile observed in the cells of origin (Figure 3A). Consequently, we observed a dramatic change in the expression profile of the cell cycle proteins involved in the G1/S transition in the ASThiPS cell lines to levels similar to those observed in hES cells (Figure 3B).

**Figure 4:**
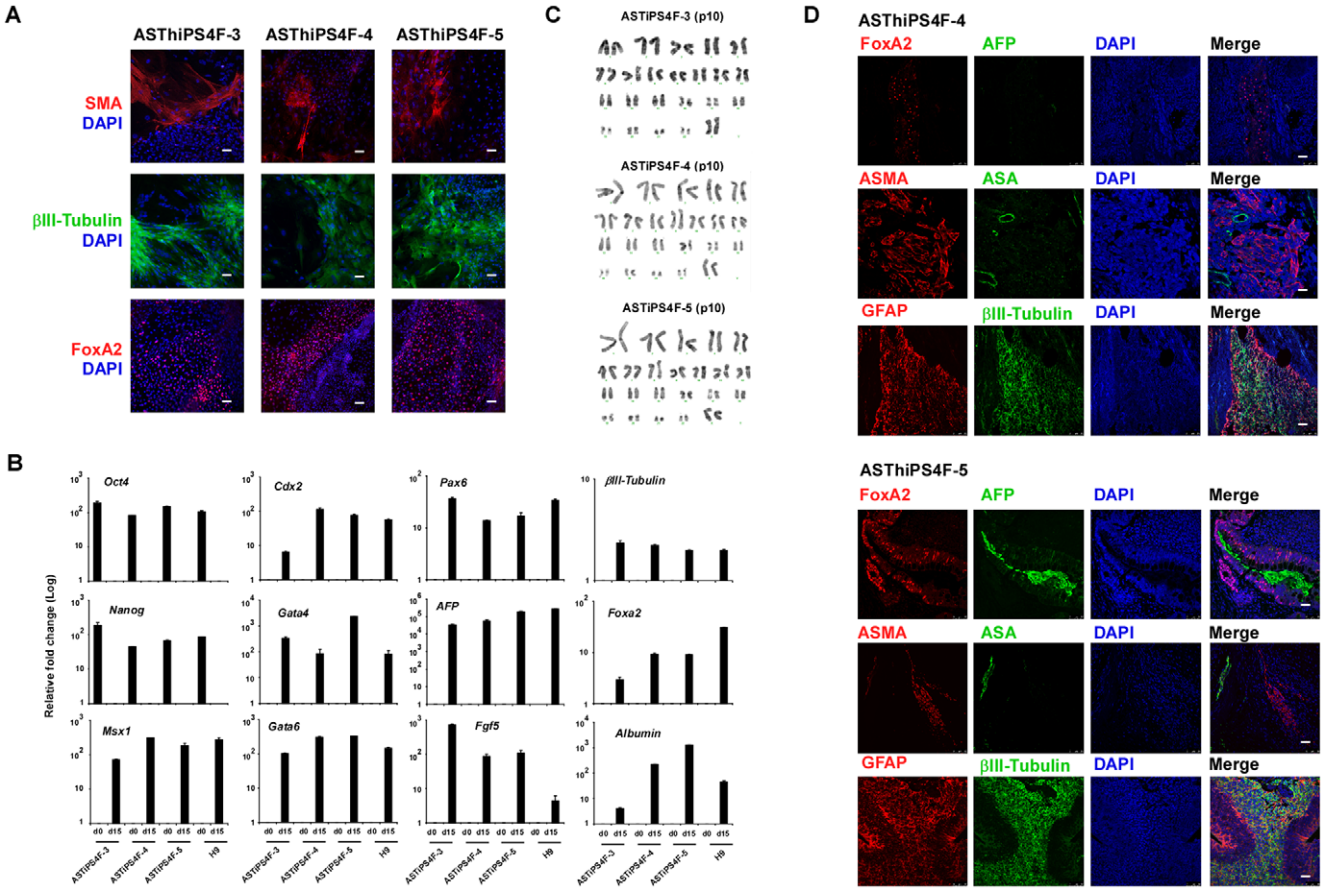
ASThiPS cell lines differentiate into the three embryonic germ layers. (A–B) Embryoid bodies were derived from ASThiPS cell lines or H9 cells maintained on MEFs, and were either collected following the initial formation (EB day 0) or following differentiation for 15 days in EB media (EB day 15). Immunofluorescence (A) and real-time PCR analysis (B) were performed for specific differentiation markers from the three embryonic germ layers as defined by the expression of mesodermal (Msx1, Smooth muscle actin (SMA)), trophectodermal (Cdx2), ectodermal (Pax6, βIIITubulin, FGF5) and endodermal (GATA4, GATA6, AFP, Albumin and FoxA2). For immuofluorescences, DAPI was used to visualize the nuclei (A). Scale bar: 50 µM. For real-time PCR, data are shown as relative average fold changes ± SEM from two biological replicates analyzed in triplicate (B). Expression of the pluripotent markers Oct4 and Nanog was assessed as a control for efficient differentiation (B). (C) G-banding karyotype analysis reveals a normal karyotype of the noted ASThiPS cell lines. The passage number at which the karyotype was analyzed is indicated in the figure. (D) Teratoma formation was assessed by injection of the ASThiPS cells into the testes or kidney of SCID mice. Immunofluorescence analysis demonstrate the existence of the three main embryonic germ layers as defined by the expression of specific endodermal (AFP (α-fetoprotein), and FoxA2), ectodermal (βIIITubulin and GFAP) and mesodermal (ASMA (alpha-smooth muscle actin) and ASA (alpha sarcomeric actin) markers. All images were obtained from the same tumor. Scale bar: 50 µM.

Finally, the pluripotency of each ASThiPS cell line was assessed by differentiation into the three embryonic germ layers *in vitro,* using embryoid body (EB) formation (Figure 4A and B), and *in vivo,* by teratoma formation (Figure 4C). Immunofluorescence analysis (Figure 4A) and real-time PCR analysis of ectodermal, endodermal and mesodermal markers following EB differentiation demonstrated that ASThiPS cells have the ability to generate all three embryonic germ layers *in vitro* (Figure 4A and B). Similarly, injection of ASThiPS cell lines into nude mice resulted in the formation of teratomas containing tissues derived from all three embryonic germ layers (Figure 4D). Finally, karyotype analysis revealed that ASThiPS cell lines had normal chromosomal integrity (Figure 4C). Thus, these combined findings demonstrate that ASThiPS cells are pluripotent.

### ASTiPS cell lines differentiate into different neural cell types

**Figure 5:**
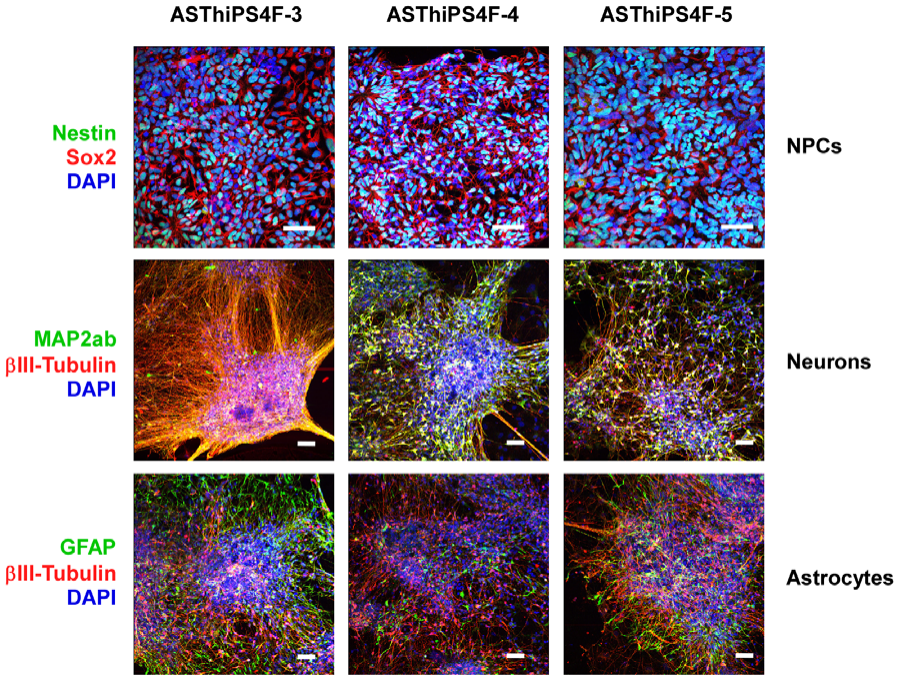
ASThiPS cell lines differentiate into Neural Progenitor Cells (NPCs), neurons and astrocytes. Top row panels: hiPS cell-derived NPCs express Nestin throughout the cytoplasm and Sox2 specifically within the nucleus. Middle row panels: hiPS-derived neurons express the neuronal marker βIII-tubulin and the dendritic marker MAP2ab. Bottom row panels: hiPS-derived astrocytes express the astrocyte marker GFAP but not the neuronal marker βIII-tubulin. DAPI was used to visualize the nuclei. Scale bar: 50 µM.

To assess the potential of ASThiPS cell lines to differentiate into neural lineages, we adopted a step-wise *in vitro* neural differentiation protocol routinely used with hES cells [28] to the ASThiPS cell lines and thereby generated ASThiPS-derived neural progenitor cells (NPCs). NPCs express Nestin and Sox2 and are capable of differentiating to mature postmitotic neurons and astrocytes [29]. Neurons express markers such as βIII-tubulin and MAP2ab: βIII-tubulin is regarded as a neuron-specific marker and its expression has been suggested to be one of the earliest markers to signal neuronal commitment in primitive neuroepithelium [30], while MAP2ab is a neuron-specific microtubule-associated protein, the expression of which is restricted to dendrites during neuronal maturation [31]. Glial Fibrillary Acidic Protein (GFAP) is a 50 kDa intracytoplasmic filamentous protein that constitutes a portion of the cytoskeleton in astrocytes and is the most specific marker for cells of astrocytic origin [32]. ASThiPS-derived NPCs express Nestin and Sox2, ASThiPS-derived neurons express both βIII-tubulin and MAP2ab, and ASThiPS-derived astrocytes express GFAP but not βIII-tubulin (Figure 5). These data confirmed the ability of ASThiPS cells to generate mature differentiated cells of neural origin.

## Discussion

The induction of pluripotency by expression of Oct4, Sox2, Klf4 and c-Myc has been reported in human somatic cells of different origin [2], [21]–[26]. Recently, the generation of iPS cells from human neural stem cells has been achieved with only the expression of Oct4 [25]. These data suggest that the high similarity in the transcriptional program reported between these committed stem cells and hES cells helps to facilitate the reprogramming process [25]. In our work, we demonstrated the ability to generate hiPS cells from a differentiated neural cell type. Furthermore, we showed that human astrocytes generated hiPS (ASThiPS) with a reprogramming efficiency similar to human keratinocytes, which is much higher than the efficiency observed for other cell types, such as fibroblasts (Figure 1). This might be due to the fact that astrocytes or keratinocytes are cell types closer to hES cells in terms of gene expression of human stem cell-related genes (Figure 1). ASThiPS cell lines expressed endogenous pluripotent markers (Figure 2), acquired the stem cell cycle signature of human ES cells (Figure 3), differentiated *in vitro* and *in vivo* into the three embryonic germ layers and displayed a normal karyotype (Figure 4). We also demonstrated the ability of the ASThiPS cell lines, using a neural directed differentiation protocol *in vitro*, to differentiate into neural lineages (i.e. neurons and astrocytes).

It has recently been reported that mesenchymal to epithelial transition (MET) and therefore, the consequent activation of an epithelial program, is an essential step in the successful reprogramming of fibroblasts, a mesenchymal cell-type [33], [34]. It is tempting to speculate that this contributes to the fact that keratinocytes and astrocytes, both epithelial-cell types, show higher reprogramming efficiencies compared to other cell types of a different origin. A detailed analysis of the reprogramming process occurring in cells of different embryonic origins might unravel the mechanisms driving the acquisition of pluripotency.

Finally, in order to apply iPS cell technology to regenerative medicine, the reprogramming process must be better understood. Towards this end, there is a need for highly efficient sources of somatic cells with which to study this process. The finding that astrocytes can be reprogrammed with high efficiency may assist in achieving this aim.

## Materials and Methods

### hES cell culture and differentiation

Human H9 (WA09) and H1 (WA01) embryonic stem cell lines were obtained from WiCell Research Institute and maintained on mitotically inactive mouse embryonic fibroblast (MEF, Millopore) feeder layers in hES cell medium: DMEM/F12 (Invitrogen) supplemented with 20% Knockout Serum Replacement (Invitrogen), 1 mM L-glutamine, 0.1 mM non-essential amino acids, 55 µM β-mercaptoethanol and 10 ng/ml bFGF (Joint Protein Central). In some cases, hES cells were also maintained on Matrigel (BD Biosciences) using either MEF-conditioned hES medium or mTeSR1 (Stem Cell Technologies). hES cell colonies were split using a solution of collagenase (1 mg/ml) or dispase (2 mg/ml) and scraping the colonies with a glass pipette. Derived hiPS cells were cultured similarly as described above for hES cells. For differentiation into embryoid bodies (EBs), hES or hiPS cell colonies growing on MEFs were loosely detached by dispase treatment, resuspended in EB media (DMEM/F12 supplemented with 10% FBS (Atlanta Biologicals), 0.5 mM L-glutamine, 0.1 mM non-essential amino acids and 55 µM β-mercaptoethanol), and maintained on low attachment plates with daily media changes. 4 days later the floating EBs were plated on gelatin-coated plates and allowed to differentiate for another 10 days in EB media. Similar experiments were performed using H1 or H9 cell lines. 293T cells were cultured in DMEM (Invitrogen) supplemented with 10% FBS and 0.1 mM non-essential aminoacids. Human keratinocytes were obtained and cultured as described [24]. Human astrocytes isolated from human cerebellar tissue were obtained from ScienCell Research Laboratories. Astrocytes were characterized by immunofluorescence with antibodies to GFAP, and are able to be expanded for 10 population doublings, as described by ScienCell Research Laboratories. Two different cellular batches of astrocytes were used in this study. IMR90 were obtained from ATCC and cultured in DMEM, 10% FBS (Atlanta Biologicals) and 0.1 mM non-essential amino acids.

### Human hiPS cell generation

For the formation of human hiPS cells, astrocytes, fibroblasts or keratinocytes were infected with an equal ratio of retroviruses encoding Oct4, Sox2, Klf4 and cMyc (OSKC) by spinfection of the cells at 1850 rpm for 1 hour at room temperature in the presence of polybrene (4 µg/ml). After two infections at day 0 and day 1, cells were passaged onto fresh MEFs and switched to hES cell medium at day 4 after the infection. Colonies were stained for either alkaline phosphatase (AP) or Nanog expression at day 18. For the derivation of hiPS cells lines, colonies were manually picked and maintained on fresh MEF feeder layers for 5 passages before growth in Matrigel/mTesR1 conditions. To calculate the efficiency of reprogramming, we first evaluated the percentage of infected cells, based on GFP expression by flow cytometry, and plated the same number of GFP^+^ cells on MEFs after the infection. Then, the final number of Nanog^+^ colonies at day 18 was evaluated by immunohistochemistry.

### hiPS differentation to NPCs and Neurons

hiPS cells growing in hES media on MEFs were incubated with collagenase (1 mg/ml in DMEM) at 37°C for 1–2 hours until colonies lifted from the plate. Colonies were washed with DMEM, transferred to a nonadherent plate (Costar) and maintained in suspension in N2 media (DMEM/F12-Glutamax, 1X N2, Invitrogen). After 7 days, EBs were plated in N2 media onto PORN/Laminin-coated plates. Visible rosettes formed within 1 week and were manually dissected onto PORN/Laminin-coated plates. Rosettes were cultured in NPC media (N2 media with 1 µg/ml laminin and 20 ng/ml FGF-2) and dissociated in Tryple (Gibco) for 3 minutes at 37°C. NPCs were maintained best at high density and split approximately 1∶5 every week. To generate neurons, NPCs were dissociated with Tryple and plated onto PORN/Laminin-coated plates in neural differentiation media (DMEM/F12-Glutamax, 1X B27-RA, 1X N2 with 20 ng/ml BDNF, 20 ng/ml GDNF (Peprotech), 1 mm dibutyrl-cyclicAMP (Sigma), 200 nm ascorbic acid (Sigma) at a density of 50,000 cells per well of a 4-well permanox slide. Neurons were analyzed following one month of differentiation of NPCs in neural differentiation media.

### Flow cytometry analysis

For BrdU detection, cells were incubated with hES cell media or mTeSR1 containing 10 µM BrdU for 30 minutes. Cells were individualized using TrypLE Express (Invitrogen) 1∶4 in PBS (Calcium^−^/Magnesium^−^) and fixed in 70% ethanol overnight. The cell pellet was resuspended in cold 0.1 M HCl/0.5% TritonX-100 for 10 minutes, after which the cells were boiled for 10 minutes in a water bath and transferred to ice for 5 minutes to cool. After a brief incubation in 0.5% Triton-X100 in PBS, cells were incubated with a rat-antiBrdU antibody (1∶100 dilution; Axyll) for 30 minutes followed by incubation with an Alexa-Fluor 488 goat anti-rat secondary antibody (Invitrogen) for 20 minutes. The cell pellet was resuspended in PBS containing 5 µg/ml of propidium iodide and 100 µg/ml of RNaseA. Flow cytometry analyses were conducted using a FACScan (BD Bioscience) and data was analyzed using FlowJo and WinMDI 2.8 software.

### RNA isolation and real time-PCR analysis

Total RNA was isolated using Trizol Reagent (Invitrogen) according to the manufacturer's recommendations, and cDNA synthesized using the SuperScript II Reverse Transcriptase kit for RT-PCR (Invitrogen). Real-time PCR was performed using the SYBR-Green PCR Master mix (Applied Biosystems). Values of gene expression were normalized using GAPDH expression and are shown as fold change relative to the value of the sample control. All the samples were done in triplicate. A list of the primers used for real time-PCR experiments are listed in Tables S1 and S2. Primers of the genes selected for the described stem cell array were based on the Human Stem RT2 Cell Array (SuperArray Biosciences Corporation) including genes involved in self-renewal, cell cycle, chromosome and chromatin modulators, cytokines and growth factors, cell communication and adhesion, metabolic markers, stem cell maintenance or asymmetric division as well as the expression of the reprogramming genes. For a complete list of the genes see Table S1.

### Plamids

pMX-Oct4, pMX-SOX2, pMX-KLF4 and pMX-cMyc were obtained from Addgene (plasmids 17217, 17218, 17219 and 17220 respectively). pMX-eGFP was kindly provided by Dr. Teruhisa Kawamura (Gene Expression Laboratory, The Salk Institute, La Jolla, CA).

### Retroviral production

Moloney-based retroviral vectors (pMX-) were co-transfected with packaging plasmids (pCMV-gag-pol-PA and pCMV-VSVg, kindly provided by Dr. Gerald Pao, Laboratory of Genetics, The Salk Institute, La Jolla, CA) in 293T cells using Lipofectamine (Invitrogen). Retroviral supernatants were collected 24 and 48 hours after transfection, pooled and passed through a 0.45 µM filter to remove cellular debris.

### Immunostainings

For the immunohistochemical detection of Nanog, cells were fixed with 4% formaldehyde in PBS for 15 minutes, washed with PBS and incubated with 0.5% Triton-X100 in PBS for 10 minutes. A rabbit anti-hNanog (1∶500) in 1% PBS-BSA was used for overnight incubation at 4°C followed by incubation with a secondary biotin-conjugated anti-rabbit antibody (1∶2000) for an additional 2 hours at room temperature (RT). Cells were then incubated with streptavidin-HRP (Vector, 1∶1000) and a DAB substrate kit for peroxidase (Vector, SK-4100) was used to develop the staining. For standard immunofluorescence, cells were fixed with 4% paraformaldehyde in PBS for 15 minutes, washed in PBS incubated with 0.5% Triton-X100 in PBS for 10 minutes and blocked with 5% normal donkey serum in 1% PBS-BSA for 1 hour at RT. Antibodies in 1% PBS-BSA (see Table S3 for details) were used for overnight incubation followed by incubation with a secondary AlexaFluor 488 or 568 (Invitrogen) for an additional 2 hours at room temperature. DAPI was used to visualize nuclei at a concentration of 10 µg/ml in PBS.

### Karyotype analysis

hiPS cell lines grown on Matrigel were cultured in the presence of 20 ng/ml of colcemid for 45 minutes. The cells were trypsinized, washed with PBS and resuspended in a hypotonic solution by drop-wise addition while vortexing at low speed. After 10 minutes of incubation at 37°C, cells were fixed by drop-wise addition of 1 ml of cold Carnoy fixative. Stained metaphases were analyzed with Cytovision software (Applied Imaging).

### Teratoma formation

Severe combined immunodeficient (NOD.Cg-*Prkdc^scid^ Il2rg^tm1Wjl^*/SzJ; Jackson Laboratories) were used to test the teratoma induction capacity of the iPS cell lines. Briefly, ∼10^6^ iPS cells in ∼50 µL of hES cell medium were injected into the testis or kidney capsule of anesthetized mice. Mice were monitored for teratoma formation and euthanized ∼6–12 weeks after injection. Teratomas were processed and analyzed by hematoxylin and eosin staining and conventional immunofluorescence. All animal experiments were performed and approved (accepted protocol number 08-025) in full accordance of The Salk Institute Institutional Animal Care and Use Committee (IACUC) guidelines.

### Western blot analysis

Cell pellets were lysed in 10 mM Tris-HCl (pH 8), 150 mM NaCl, 1% Triton X100, 1 mM Na_3_VO_4_, 1 mM PMSF and the Cømplete protease inhibitor mixture (Roche). Total protein extracts (25 µg) were used for SDS-PAGE, transferred to nitrocellulose membranes (Amersham Biosciences) and analyzed using primary antibodies (see Table S3 for details). Horseradish peroxidase-conjugated secondary anti-mouse or rabbit were purchased from Cell Signaling and used at 1∶5000 dilution. Tubulin was used as a loading control. Immunoblots were visualized using SuperSignal solutions following the manufacturer's instructions (Thermo Scientific).

### Alkaline phosphatase (AP) staining

For AP staining, cells were fixed in a solution of 4% paraformaldehyde in PBS for 20 minutes. After extensive washes in PBS, cells were incubated in NTMT solution (10 mM NaCl, 100 mM Tris-HCl (pH 9), 50 mM MgCl2 and 0.1% Tween-20) for 5 minutes and then in NTMT solution supplemented with NBT (Nitro-Blue Tetrazolium Chloride) and BCIP (5-Bromo-4-Chloro-3'-Indolyphosphate p-Toluidine Salt) in the dark until the staining developed.

### Statistical analysis

Results are reported as an average ± SEM (see figure legends for specific details regarding the number of biological replicates, independent experiments and technical replicates). Statistics were performed using two-tailed T-student analyses. Values with p<0.05 were considered statistically significant. The values obtained from the stem cell array were clustered using a Pearson Correlation.

## Supporting Information

Table S1 Forward and reverse sequence of the primers used for the described stem cell array which include genes involved in selfrenewal, cell cycle, chromosome and chromatin modulators, cytokines and growth factors, cell communication and adhesion, metabolic markers, stem cell maintenance or asymmetric division as well as the expression of the reprogramming genes.
(DOC)

Table S2 Forward and reverse sequence of the primers used in this study to analyze the expression of pluripotent genes, differentiation markers and cell cycle regulators.
(DOC)

Table S3 List of the antibodies used in this study. IF: Immunofluorescence. WB: Western Blot. Flow Cyt: Flow Cytometry.
(DOC)
