# Supplementary material for: High-Efficient Generation of Induced Pluripotent Stem Cells from Human Astrocytes
Source: PLoS One. 2010 Dec 9;5(12):e15526. doi: 10.1371/journal.pone.0015526 (PMC3000364; doi:10.1371/journal.pone.0015526)
Supplement: Table S1 — Forward and reverse sequence of the primers used for the described stem cell array which include genes involved in selfrenewal, cell cycle, chromosome and chromatin modulators, cytokines and growth factors, cell communication and adhesion, metabolic markers, stem cell maintenance or asymmetric division as well as the expression of the reprogramming genes. (DOC) [file pone.0015526.s001.doc]

**Table S1: List of primers (stem cell profiler):**

| **Primer** | **Sequence (5’ to 3’)** | **Application** |
| --- | --- | --- |
| h-APC-F | GGAGACAGAATGGAGGTGCT | qPCR |
| h-APC-R | TCTTCAGTGCCTCAACTTGC | qPCR |
| h-Axin1-F | CCGGCATTGACATAATAGGG | qPCR |
| h-Axin1-R | CCGAGGGTCTCCTCCAGTA | qPCR |
| h-EP300-F | TCTGGTAAGTCGTGCTCCAA | qPCR |
| h-EP300-R | GCGGCCTAAACTCTCATCTC | qPCR |
| h-FGF1-F | TGTGGAGAGAGGTACAGCCC | qPCR |
| h-FGF1-R | AAGGTGGTGATTTCCCCTTC | qPCR |
| h-FGF2-F | CCTCTCTCTTCTGCTTGAAGTTG | qPCR |
| h-FGF2-R | AGCGGCTGTACTGCAAAAAC | qPCR |
| h-FGF3-F | ATTATAGCCCAGCTCGTGGA | qPCR |
| h-FGF3-R | TACCTGGCCATGAACAAGAG | qPCR |
| h-FGF4-F | GAAGATGCTCACCACGCC | qPCR |
| h-FGF4-R | CATCGGCTTCCACCTCC | qPCR |
| h-Notch2-F | CACAGGGTTCATAGCCATCTC | qPCR |
| h-Notch2-R | GGAGGCGACCGAGAAGAT | qPCR |
| h-PARD6a-F | AGCAACCGCGAGAACTCC | qPCR |
| h-PARD6a-R | GCGCAGTCCCGATAGCAT | qPCR |
| h-GCN5L2-F | CGGCGTAGGTGAGGAAGTAG | qPCR |
| h-GCN5L2-R | GTGCTGTCACCTCGAATGAG | qPCR |
| h-HDAC2-F | ATGAGGCTTCATGGGATGAC | qPCR |
| h-HDAC2-R | ATGGCGTACAGTCAAGGAGG | qPCR |
| h-MYST1-F | CCTCATGCTCCTTCTCCAAG | qPCR |
| h-MYST1-R | GATCACTCGCAACCAAAAGC | qPCR |
| h-MYST2-F | TGATACAGTGGGCATCCTGA | qPCR |
| h-MYST2-R | TGAAGTGTCCTACACCAGGC | qPCR |
| h-TERT-F | CAGGATCTCCTCACGCAGAC | qPCR |
| h-TERT-R | GAGCTGACGTGGAAGATGAG | qPCR |
| h-DHH-F | ACATGTTCATCACGGCAATG | qPCR |
| h-DHH-R | ACCTCGTGCCCAACTACAAC | qPCR |
| h-Notch1-F | ATAGTCTGCCACGCCTCTG | qPCR |
| h-Notch1-R | AGTGTGAAGCGGCCAATG | qPCR |
| h-Numb-F | GGCCCACCAATATTCCAATC | qPCR |
| h-Numb-R | GTGGCGCTTGAGTTGGTC | qPCR |
| h-Neurog1-F | ATCCGAGCAGCACTAACACG | qPCR |
| h-Neurog1-R | GCACAGGCCAAAGTCACAG | qPCR |
| h-BMP1-F | GGGACGTGAAGTTCAGGATG | qPCR |
| h-BMP1-R | TTCTCCTCCCCTGAATACCC | qPCR |
| h-BMP2-F | CCTCCGTGGGGATAGAACTT | qPCR |
| h-BMP2-R | CACTGTGCGCAGCTTCC | qPCR |
| h-BMP3-F | TCAGGCTGATGTTTCCTAGC | qPCR |
| h-BMP3-R | ACACGGTTCGCAGCTTTC | qPCR |
| h-cxcl12-F | TGGGCTCCTACTGTAAGGGTT | qPCR |
| h-cxcl12-R | TTGACCCGAAGCTAAAGTGG | qPCR |
| h-gdf2-F | AAGATGTGCTTCTGGAAGGG | qPCR |
| h-gdf2-R | TCCGATAAGTCGACTACGCC | qPCR |
| h-gdf3-F | TGCTACGTAAAGGAGCTGGG | qPCR |
| h-gdf3-R | CAGGAGGAAGCTTGGGAAAT | qPCR |
| h-igf1-f | TCATCCACGATGCCTGTCT | qPCR |
| h-igf1-r | TGGATGCTCTTCAGTTCGTG | qPCR |
| h-jag1-f | CTGTCAGGTTGAACGGTGTC | qPCR |
| h-jag1-R | CTTCAACCTCAAGGCCAGC | qPCR |
| h-dll1-F | CAGGGTTGCACACTTTCTCC | qPCR |
| h-dll1-R | ACTCCTACCGCTTCGTGTGT | qPCR |
| h-gja1-F | GAGTTTGCCTAAGGCGCTC | qPCR |
| h-gja1-R | AGGAGTTCAATCACTTGGCG | qPCR |
| h-gjb1-f | CTGTCCAGTTCATCCTGCCT | qPCR |
| h-gjb1-r | CCTGCACAGACATGAGACCA | qPCR |
| h-gjb2-F | GGTGGAGTGTTTGTTCACACC | qPCR |
| h-gjb2-R | AGCGCAGAGACCCCAAC | qPCR |
| h-bgla-F | TTGGACACAAAGGCTGCAC | qPCR |
| h-bgla-R | CTCACACTCCTCGCCCTATT | qPCR |
| h-Cd4-F | CTTGGTCCCAAAGGCTTCTT | qPCR |
| h-Cd4-R | AGATTCTGGGAAATCAGGGC | qPCR |
| h-Cd44-F | CACGTGGAATACACCTGCAA | qPCR |
| h-Cd44-R | GACAAGTTTTGGTGGCACG | qPCR |
| h-cdh1-F | GACCGGTGCAATCTTCAAA | qPCR |
| h-cdh1-R | TTGACGCCGAGAGCTACAC | qPCR |
| h-cdh2-F | CCACCTTAAAATCTGCAGGC | qPCR |
| h-cdh2-R | GTGCATGAAGGACAGCCTCT | qPCR |
| h-col9a1-F | GGGATCCCACTGGTCCTAAT | qPCR |
| h-col9a1-R | GTCAGATGGGAAATTCAGGC | qPCR |
| h-ctnna1-F | GTCCCTGGTCTTCTTGGTCA | qPCR |
| h-ctnna1-R | AGAGTGTAATGCTGTCCGCC | qPCR |
| h-NCAN1-F | TGGCTGGGAACAATATCCAC | qPCR |
| h-NCAN1-R | CAGCCAGCAGATTACAATGC | qPCR |
| h-ABCG2-F | TGGTGTTTCCTTGTGACACTG | qPCR |
| h-ABCG2-R | TGAGCCTTTGGTTAAGACCG | qPCR |
| h-aldh1-F | CCACTCACTGAATCATGCCA | qPCR |
| h-aldh1-R | GCACGCCAGACTTACCTGTC | qPCR |
| h-aldh2-F | CCTCTCCAGTGGACGGATT | qPCR |
| h-aldh2-R | CGAGGTCTTCTGCAACCAG | qPCR |
| h-fgfr1-F | GGAAGGACTCCACTTCCACA | qPCR |
| h-fgfr1-R | GTCACAGCCACACTCTGCAC | qPCR |
| h-agc1-F | ACAGCTGCAGTGATGACCCT | qPCR |
| h-agc1-R | TTCTTGGAGAAGGGAGTCCA | qPCR |
| h-alpi-F | TCAGCTGGGTACTCAGGGTC | qPCR |
| h-alpi-R | ATCGCCACTCAGCTCATCTC | qPCR |
| h-col1a1-F | AAGAGGAAGGCCAAGTCGAG | qPCR |
| h-col1a1-R | CACACGTCTCGGTCATGGTA | qPCR |
| h-col2a1-F | CGGCTTCCACACATCCTTAT | qPCR |
| h-col2a1-R | CTGTCCTTCGGTGTCAGGG | qPCR |
| h-pparg-F | GAGAGATCCACGGAGCTGAT | qPCR |
| h-pparg-R | AGGCCATTTTGTCAAACGAG | qPCR |
| h-dll3-F | CCTGCGCGCTGAATGTC | qPCR |
| h-dll3-R | CATCGAAACCTGGAGAGAGG | qPCR |
| h-dtx1-F | ACGACGGGTCGTAGAAGTTG | qPCR |
| h-dtx1-R | GCTTGTGCCCTACATCATCG | qPCR |
| h-dtx2-F | CTGCTTTCCCAAACAGCTTC | qPCR |
| h-dtx2R | GGGTCCCCAAAGAAGTCAAG | qPCR |
| h-dvl1-F | GTGTGATCCGATTCACTGCC | qPCR |
| h-dvl1-R | CGGGAGTCAGCAGAGTGAAG | qPCR |
| h-adar-F | CTTGCCTTGCTTCTTGCTGT | qPCR |
| h-adar-R | TGCTGAATTCAAGTTGGTCG | qPCR |
| h-btrc-F | CTAGGGGGTTCGCCATTATT | qPCR |
| h-btrc-R | AGTGGCCTCGGCGATTAT | qPCR |
| h-frat1-F | CTTCTCCATTTGGAGGGTCA | qPCR |
| h-frat1-R | GCAATTCCCTGGATAGACGA | qPCR |
| h-fzd1-F | CAGCACAGCACTGACCAAAT | qPCR |
| h-fzd1-R | GTGAGCCGACCAAGGTGTAT | qPCR |
| h-ppard-F | TGAACGCAGATGGACCTCTA | qPCR |
| h-ppard-R | TCACACAGTGGCTTCTGCTC | qPCR |
| h-wnt1-F | GGAGGAGGCTACGTTCACAA | qPCR |
| h-wnt1-R | TTTCTGCTACGCTGCTGCT | qPCR |
| h-E2F1-F | TGCAGAGCAGATGGTTATGG | qPCR |
| h-E2F1-R | CTGATCCCACCTACGGTCTC | qPCR |
| h-CycD1-F | CCCTCGGTGTCCTACTTCAA | qPCR |
| h-CycD1-R | TCCTCGCACTTCTGTTCCTC | qPCR |
| h-CycD2-F | GTCTCAAAGCTTGCCAGGAG | qPCR |
| h-CycD2-R | ATATCCCGCACGTCTGTAGG | qPCR |
| h-p16-F | GAGCAGCATGGAGCCTTCG | qPCR |
| h-p16-R | CATCATCATGACCTGGATCG | qPCR |
| h-p21-F | GGAAGACCATGTGGACCTGT | qPCR |
| h-p21-R | GGCGTTTGGAGTGGTAGAAA | qPCR |
| h-CycE1-F | CGGTATATGGCGACACAAGA | qPCR |
| h-CycE1-R | ACATACGCAAACTGGTGCAA | qPCR |
| h-CycA2-F | CCTGCAAACTGCAAAGTTGA | qPCR |
| h-CycA2-R | AAAGGCAGCTCCAGCAATAA | qPCR |
| h-Msx1-F | CTCCGCAAACACAAGACGAAC | qPCR |
| h-Msx1-R | CACATGGGCCGTGTAGAGTC | qPCR |
| h-Tubb3-F | CCTGGAACCCGGAACCAT | qPCR |
| h-Tubb3-R | AGGCCTGAAGAGATGTCCAAAG | qPCR |
| h-FoxA1-F | CCAAGGCCGCCTTACTCCTACA | qPCR |
| h-FoxA1-R | CGCAGATGAAGACGCTTGGAGA | qPCR |
| h-Oct4-end-F | GGGTTTTTGGGATTAAGTTCTTCA | qPCR |
| h-Oct4-end-R | GCCCCCACCCTTTGTGTT | qPCR |
| h-Sox2-end-F | CAAAAATGGCCATGCAGGTT | qPCR |
| h-Sox2-end-R | AGTTGGGATCGAACAAAAGCTATT | qPCR |
| h-Klf4-end-F | AGCCTAATTGATGGTGCTTGGT | qPCR |
| h-Klf4-end-R | TTGAAAACTTTGGCTTCCTTGTT | qPCR |
| h-cMyc-end-F | CGGGCGGGCACTTTG | qPCR |
| h-cMyc-end-R | GGAGAGTCGCGTCCTTGCT | qPCR |
| h-p15-F | CGGGGACTAGTGGAGAAGGT | qPCR |
| h-p15-R | CCCATCATCATGACCTGGAT | qPCR |
| h-GAPDH-F | GGACTCATGACCACAGTCCATGCC | qPCR |
| h-GAPDH-R | TCAGGGATGACCTTGCCCACAG | qPCR |
