# Supplementary material for: High-Efficient Generation of Induced Pluripotent Stem Cells from Human Astrocytes
Source: PLoS One. 2010 Dec 9;5(12):e15526. doi: 10.1371/journal.pone.0015526 (PMC3000364; doi:10.1371/journal.pone.0015526)
Supplement: Table S2 — Forward and reverse sequence of the primers used in this study to analyze the expression of pluripotent genes, differentiation markers and cell cycle regulators. (DOC) [file pone.0015526.s002.doc]

**Table S2: List of primers:**

| **Primer** | **Sequence (5’ to 3’)** | **Application** |
| --- | --- | --- |
| h-E2F1-F | TGCAGAGCAGATGGTTATGG | qPCR |
| h-E2F1-R | CTGATCCCACCTACGGTCTC | qPCR |
| h-CycD1-F | CCCTCGGTGTCCTACTTCAA | qPCR |
| h-CycD1-R | TCCTCGCACTTCTGTTCCTC | qPCR |
| h-CycD2-F | GTCTCAAAGCTTGCCAGGAG | qPCR |
| hCycD2-R | ATATCCCGCACGTCTGTAGG | qPCR |
| h-CycD3-F | TGACCATCGAAAAACTGTGC | qPCR |
| h-CycD3-R | GAATGAAGGCCAGGAAATCA | qPCR |
| h-p16-F | GAGCAGCATGGAGCCTTCG | qPCR |
| h-p16-R | CATCATCATGACCTGGATCG | qPCR |
| h-p21-F | GGAAGACCATGTGGACCTGT | qPCR |
| h-p21-R | GGCGTTTGGAGTGGTAGAAA | qPCR |
| h-p18-1F | CGTCAATGCACAAAATGGA | qPCR |
| h-p18-1R | CGAAACCAGTTCGGTCTTTC | qPCR |
| h-CycE1-F | CGGTATATGGCGACACAAGA | qPCR |
| h-CycE1-R | ACATACGCAAACTGGTGCAA | qPCR |
| h-CycA2-F | CCTGCAAACTGCAAAGTTGA | qPCR |
| h-CycA2-R | AAAGGCAGCTCCAGCAATAA | qPCR |
| h-GATA4-F | ACACCCCAATCTCGATATGTTTG | qPCR |
| h-GATA4-R | GTTGCACAGATAGTGACCCGT | qPCR |
| h-GATA6-F | AGGGCTCGGTGAGTCCAAT | qPCR |
| h-GATA6-R | CGCTGCTGGTGAATAAAAAGGA | qPCR |
| h-cdx2-F | GGCAGCCAAGTGAAAACCAG | qPCR |
| h-cdx2-R | GGTGATGTAGCGACTGTAGTGAA | qPCR |
| h-AFP-F | ACTGAATCCAGAACACTGCATAG | qPCR |
| h-AFP-R | GCTTCTTGAACAAACTGGGCAAA | qPCR |
| h-Pax6-F | ACAGTCACAGCGGAGTGAATC | qPCR |
| h-Pax6-R | ACTTTTGCATCTGCATGGGTC | qPCR |
| h-Msx1-F | CTCCGCAAACACAAGACGAAC | qPCR |
| h-Msx1-R | CACATGGGCCGTGTAGAGTC | qPCR |
| h-Tubb3-F | CCTGGAACCCGGAACCAT | qPCR |
| h-Tubb3-R | AGGCCTGAAGAGATGTCCAAAG | qPCR |
| h-FGF5-F | ATTTGCTGTGTCTCAGGGGAT | qPCR |
| h-FGF5-R | CTGTGAACTTGGCACTTGCAT | qPCR |
| h-FoxA1-F | CCAAGGCCGCCTTACTCCTACA | qPCR |
| h-FoxA1-R | CGCAGATGAAGACGCTTGGAGA | qPCR |
| h-Albumin-F | GAGTGAGGTTGCTCATCGGTTT | qPCR |
| h-Albumin-R | GCAATCAACACCAAGGCTTTG | qPCR |
| h-Oct4-end-F | GGGTTTTTGGGATTAAGTTCTTCA | qPCR |
| h-Oct4-end-R | GCCCCCACCCTTTGTGTT | qPCR |
| h-Sox2-end-F | CAAAAATGGCCATGCAGGTT | qPCR |
| h-Sox2-end-R | AGTTGGGATCGAACAAAAGCTATT | qPCR |
| h-Klf4-end-F | AGCCTAATTGATGGTGCTTGGT | qPCR |
| h-Klf4-end-R | TTGAAAACTTTGGCTTCCTTGTT | qPCR |
| h-cMyc-end-F | CGGGCGGGCACTTTG | qPCR |
| h-cMyc-end-R | GGAGAGTCGCGTCCTTGCT | qPCR |
| h-Nanog-F | ACAACTGGCCGAAGAATAGCA | qPCR |
| h-Nanog-R | GGTTCCCAGTCGGGTTCAC | qPCR |
| h-UTF1-F | CGCCGCTACAAGTTCCTTAAA | qPCR |
| h-UTF1-R | GGATCTGCTCGTCGAAGGG | qPCR |
| h-Dppa4-F | TCCTGGGCGAGAATTTCAGC | qPCR |
| h-Dppa4-R | GCAGGTGAACCCAACCATCT | qPCR |
| h-Zpf42-F | ACCGGGCAAAGACAAGACAC | qPCR |
| h-Zpf42-R | GCTGACAGGTTCTATTTCCGC | qPCR |
| h-Dppa2-F | CTGGTGCCAGTTAAAGATGACG | qPCR |
| h-Dppa2-R | TGTGGAGCTGTAAATTGCTCATT | qPCR |
| h-GAPDH-F | GGACTCATGACCACAGTCCATGCC | qPCR |
| h-GAPDH-R | TCAGGGATGACCTTGCCCACAG | qPCR |
