# Supplementary material for: High-Efficient Generation of Induced Pluripotent Stem Cells from Human Astrocytes
Source: PLoS One. 2010 Dec 9;5(12):e15526. doi: 10.1371/journal.pone.0015526 (PMC3000364; doi:10.1371/journal.pone.0015526)
Supplement: Table S3 — List of the antibodies used in this study. IF: Immunofluorescence. WB: Western Blot. Flow Cyt: Flow Cytometry. (DOC) [file pone.0015526.s003.doc]

**Table S3: List of antibodies:**

| **Protein** | **Supplier** | **Catalog number** | **Use** | **Dilution** |
| --- | --- | --- | --- | --- |
| Oct4 | Santa Cruz Biotech | sc-5279 | WB, IF | 1:500, 1:1000 |
| Sox2 | Chemicom | AB5603 | WB, IF | 1:1000, 1:500 |
| Nanog | Abcam | Ab21624 | WB, IF | 1:1000, 1:500 |
| AFP | DAKO | A0008 | IF | 1:800 |
| FoxA2 | R&D | AF2400 | IF | 1:300 |
| Tuj1 | Covance | MMS-435-P | IF | 1:500 |
| Alpha-sarcomeric actin (ASA) | Sigma | A2172 | IF | 1:500 |
| Alpha-smooth muscle actin (ASMA) | Sigma | A5228 | IF | 1:1000 |
| Tra-1-60 | BD Pharmigen | 560173 | IF | 1:100 |
| Tra-1-81 | Stemgent | 09-0069 | IF | 1:100 |
| Nestin | Chemicon | MAB5326 | IF | 1:200 |
| Sox2 | Santa Cruz | SC-17320 | IF | 1:200 |
| MAP2ab | Sigma | M1406 | IF | 1:200 |
| βIII-Tubulin | Covance | PRB435P | IF | 1:200 |
| GFAP | Dako | AB1980 | IF | 1:200 |
| BrdU | Axyll | OBT0030 | Flow Cyt | 1:100 |
| Tubulin | Sigma | T5168 | WB | 1:3000 |
